# Evaluation and management of fungal-infected carrot seeds

**DOI:** 10.1038/s41598-020-67907-5

**Published:** 2020-07-02

**Authors:** Xue Zhang, Ruiting Wang, Hailong Ning, Wenxia Li, Yunlong Bai, Yonggang Li

**Affiliations:** 1 Agricultural College, Northeast Agricultural University, Harbin, 150030 China; 2 Shuangyashan Dong Hao Agricultural Science and Technology Development Co., Ltd, Shuangyashan, 155100 China; 3 Haotian Vegetable and Corn Farmers’ Specialized Cooperative Agency in Sihe Village, Taibao Town, Sifangtai District, Shuangyashan, 155100 China

**Keywords:** Isolation, separation and purification, Seed development

## Abstract

Carrot (*Daucus carota* L.), which is one of the 10 most important vegetable crops worldwide, is an edible root vegetable desired for its taste as well as its medicinal uses. However, a fungus isolated from carrot seeds was observed to substantially decrease the germination rate. The isolate was identified as *Alternaria alternata* based on morphological and molecular characteristics as well as a phylogenetic tree*.* The maximum seed infection rate of selected carrot cultivars was approximately 60%, with the main infection site just underneath the seed shell. Additionally, the germination rate of infected seeds decreased by 28.7%. However, the seed infection rate varied among the examined carrot cultivars. Regarding the effects of chemical fungicides, the optimal treatment involved immersing seeds in amistar top suspension concentrate (SC) (effective concentration of 0.65 g/L) for 6 h, which effectively killed the fungi inside the carrot seeds. The results of this study provide a theoretical basis for the development of efficient methods for preventing the infection of carrot seeds by specific fungi and increasing the germination rate and vigour index.

## Introduction

Carrot (*Daucus carota* L.), which is cultivated worldwide, is one of the most important root vegetables in the family Apiaceae^1^. The importance of carrots in fulfilling the nutritional needs of communities has long been known^2^. In addition to being an edible root vegetable with a desirable taste, carrots have a medicinal use. The largest carrot producer worldwide is China, wherein the vegetable is cultivated mainly in the northern, northeastern, central, and southwestern parts of the country to generate 43% of the global carrot yield^3^.

Current studies on carrots mainly focus on cultivation, breeding, tissue culture, nutrient content, increasing yield, and regulating carotenoid synthesis^1,4,5^. There has been comparatively less research regarding fungal infections of seeds. We recently revealed that some carrot varieties have a low germination rate and a weak seedling growth potential. Preliminary analyses have suggested that these characteristics may be related to the infection of seeds by specific fungi. Seeds form the basis of crop production and are vital for plant associations with microorganisms, which may be damaging to the seeds or the seedlings that germinate from infected seeds^6^. Of the 16% annual crop loss due to plant diseases, at least 10% is caused by seed-borne diseases^7^.

During seed production, storage, and transport, the seeds are exposed to many kinds of microorganisms, ultimately resulting in fungal infections that may adversely affect seeds by decreasing germination and vigour, shortening the storage period, and inducing physiological changes^7,8^. Seeds infected by fungi may survive for 5 years if they are air-dried and stored at 4 °C^9^. However, seed quality directly determines the quality of agricultural products^10^. Additionally, seed-borne pathogens can be the primary source of infection and disease transmission^11^.

Fungi infecting seeds can attach to the seed surface or penetrate the seeds. Consequently, the effectiveness of chemical seed treatments may vary because deep-seated infections may be unaffected^12^. The application of chemical pesticides is a fundamental agronomic practice regarding crop protection^13^. However, their excessive use may decrease the sensitivity of the target pathogens to these chemicals. Therefore, developing novel fungicides with low toxicity and establishing appropriate application protocols for managing fungal infections of seeds are crucial. For example, Sudisha et al. (2006) revealed that a carbendazim wettable powder, captan 50 WP, and dithane M-45 can inhibit melon stem blight due to infected seeds^14^. Wang et al. observed that treatments with 75% chlorothalonil, 50% thiram, or 80% carbendazim are significantly inhibitory to the pathogens infecting seeds, and that 75% chlorothalonil and 80% carbendazim can promote seed germination^15^. Thus, research involving the screening and use of chemical fungicides may result in effective methods for controlling fungal infections of seeds.

The aim of the current study was to isolate the fungi associated with carrot seeds, evaluate the efficacy of chemical fungicides against the detected fungi, and optimize the application of fungicides to maintain a relatively high germination rate and vigour index.

## Materials and methods

### Isolation and identification of fungi infecting carrot seeds

The tested carrot seeds (cultivars Kaixinmintu and Berlin) were purchased from a commercial market in Shuangyashan, Heilongjiang province, China. The seeds (30 seeds/cultivar) were surface-sterilized with 0.5% NaOCl for 5 min, rinsed three times with sterilized distilled water, placed on potato dextrose agar (PDA), and incubated at 26 °C. Single spores were obtained from the fungal cultures for morphological and molecular analyses as previously described^16^. The fungi were identified based on morphological characteristics as described in published methods^17,18,19^. To further identify the fungi, genomic DNA was extracted from the isolates with the Fungal Genomic DNA Kit (Tiangen, Beijing, China), after which it was amplified by PCR with the universal fungal primers ITS1 and ITS4^20^ and EF1-728F/EF1-986R^21^. The PCR amplification was conducted in a 50 µl solution consisting of 25 μl *Taq* mixture (Promega, Madison, WI, USA), 2 µl ITS1 primer (10 μM), 2 µl ITS4 primer (10 μM), 2.0 µl DNA template, and 19 µl ddH_2_O. The PCR was completed with the T100™ thermocycler (Bio-Rad Laboratories Inc, CA) and the following program: 94 °C for 5 min; 36 cycles of 94 °C for 1 min, 58 °C for 1 min, and 72°C for 1.5 min; 72 °C for 10 min. The amplicons were purified and sequenced by Shanghai Biological Engineering Co., Ltd. (Shanghai, China).

### Effects of fungal infections on carrot seed germination

Carrot seeds (cv. Hanhong) were collected to analyse the impact of fungal infections on germination. amistar top SC (325 g/L) was diluted to 0.65 g/L (effective concentration), after which carrot seeds were immersed in the diluted fungicide solution for 8 h to kill all of the fungi within the seeds. Control seeds were treated with sterile water. All seeds were then air-dried and analysed. The seed infection and germination rates were determined by culturing on PDA. Specifically, the seeds were sterilized, after which 30 seeds per treatment were placed on PDA medium and incubated at 25 °C for 5 days. The appearance of the resulting fungal colonies was recorded. This analysis was completed with three replicates for a total of 90 seeds, which were used to calculate the seed infection and germination rates with the following equations:

$$ \begin{gathered} {\text{Seed infection rate }}\left( \% \right) \, = {\text{ Number of seeds infected by fungi}}/{\text{Total number of seeds }} \times { 1}00; \hfill \\ {\text{Seed germination rate}}\left( \% \right) \, = {\text{ Number of germinated seeds}}/{\text{Total number of seeds }} \times { 1}00 \hfill \\ \end{gathered} $$

### Carrot seed parts infected by fungi

Carrot seeds (cultivar Hanhong) were surface-sterilized with three replicates for a total of 90 seeds. The 90 seeds were cut in the middle with sterilized scissors and the halved seeds were placed on PDA medium. Additionally, 90 intact seeds were placed on PDA medium. After a 5-day incubation at 25 °C, the appearance of the fungal colonies was recorded. The seed infection rate was calculated to evaluate the seed parts infected by fungi.

### Determination of the seed infection rate of 10 carrot cultivars

The following 10 carrot cultivars were analysed: Hanhong (Beijing Baikutian Seedling Co., Ltd., China); Zhimeihong, Qiuhongyun, Hongyu 6, Kaixinmingtu, Kaixin666, Kaixin668, Kaixin838, and Kaixin1 (Qingdao Yuanshengtai Seed Industry Co., Ltd., China); and Berlin (Bejo Zaden B.V., Harenkarspel BEJO, Harenkarspel, The Netherlands).

The tested carrot seeds were surface-sterilized. For each carrot cultivar, 30 seeds per cultivar were placed on PDA medium, with three replicates for a total of 90 seeds per cultivar. The seeds were incubated at 26 °C for 5 days, after which the seed infection rate was calculated with the formula provided above and compared among the 10 cultivars.

### Screening of chemical fungicides

Carrot seeds (cultivar Hanhong) were treated with the following fungicides: azoxystrobin (250 g/L SC) (Shanghai Future Industry Co., Ltd., Shanghai, China); boscalid (50% WG) and sercadis plus (12% SC) [BASF Plant Protection (Jiangsu) Co., Ltd., Rudong, China]; difenoconazole (10% WG), fipronil (25 g/L DS), amistar top SC (325 g/L SC), and procymidone (18.7% SC) [Syngenta (Nantong) Crop Protection Co., Ltd., Nantong, China]; carbendazim (80% WP) (Shandong Haier Sanli Biochemical Co., Ltd., Zhucheng, China); and captan (80% WG) (Adama Makhteshim Ltd., Beersheba, Israel).

The chemical fungicides were diluted for an effective concentration of 0.65 g/L. Carrot seeds (180 seeds/fungicide) were immersed in the diluted solutions or sterile water (control) for 6 h. After air-drying the seeds, 90 of them were added to PDA medium to assess the effects of the fungicides on the seed infection rate. This analysis was completed with three replicates for a total of 90 seeds. The remaining 90 seeds were added to pots containing sterilized soil, with 10 seeds per pot and nine pots per treatment. The seeds were covered with 1 cm of soil. After a 15-day incubation in the greenhouse, the germination rate, seedling height, and fresh weight were determined. The seed infection rate was calculated with the formula provided above. The following formula was used to calculate the control effect:

$${\text{Control effect }}\left( \% \right) = ({\text{seed infection rate of thecontrol group}} - {\text{seed infection rate of thetreatment group}})/{\text{seed infection rate of thecontrol group}} \times {1}00$$

### Optimizing fungicide applications on carrot seeds

Fungicide applications were optimized, including the amistar top SC concentration and treatment time. Specifically, carrot seeds (90 seeds/treatment) were treated with amistar top SC (effective concentrations of 0.33, 0.41, or 0.65 g/L) or sterile water (control) for 6 h, after which 30 seeds per treatment were surface-sterilized and added to PDA medium (10 seeds/plate) and incubated at 26 °C. The remaining 90 seeds were added to a culture bowl (10 seeds/bowl). The seeds were incubated for 20 days in a greenhouse with a 28 °C (day): 22 °C (night) cycle. The germination rate, plant height, and fresh weight of the carrot seedlings were determined.

Sterilized carrot seeds (90 seeds/treatment) were immersed in amistar top SC solution (effective concentration of 0.65 g/L) or sterile water (control) for 2, 4, 6, or 8 h. The seed infection rate was calculated with the formula provided above. Additionally, the effect of the treatment on seedling growth was assessed.

### Statistical analysis

All experiments were conducted twice under similar conditions. Data underwent an analysis of variance with the SPSS Statistics 19.0 program (IBM/SPSS, Chicago, IL, USA). The significance of any differences in the mean values for treatments was determined with Duncan’s multiple range test (*P* < 0.05).

## Results

### Isolation and identification of fungi infecting carrot seeds

**Figure 1:**
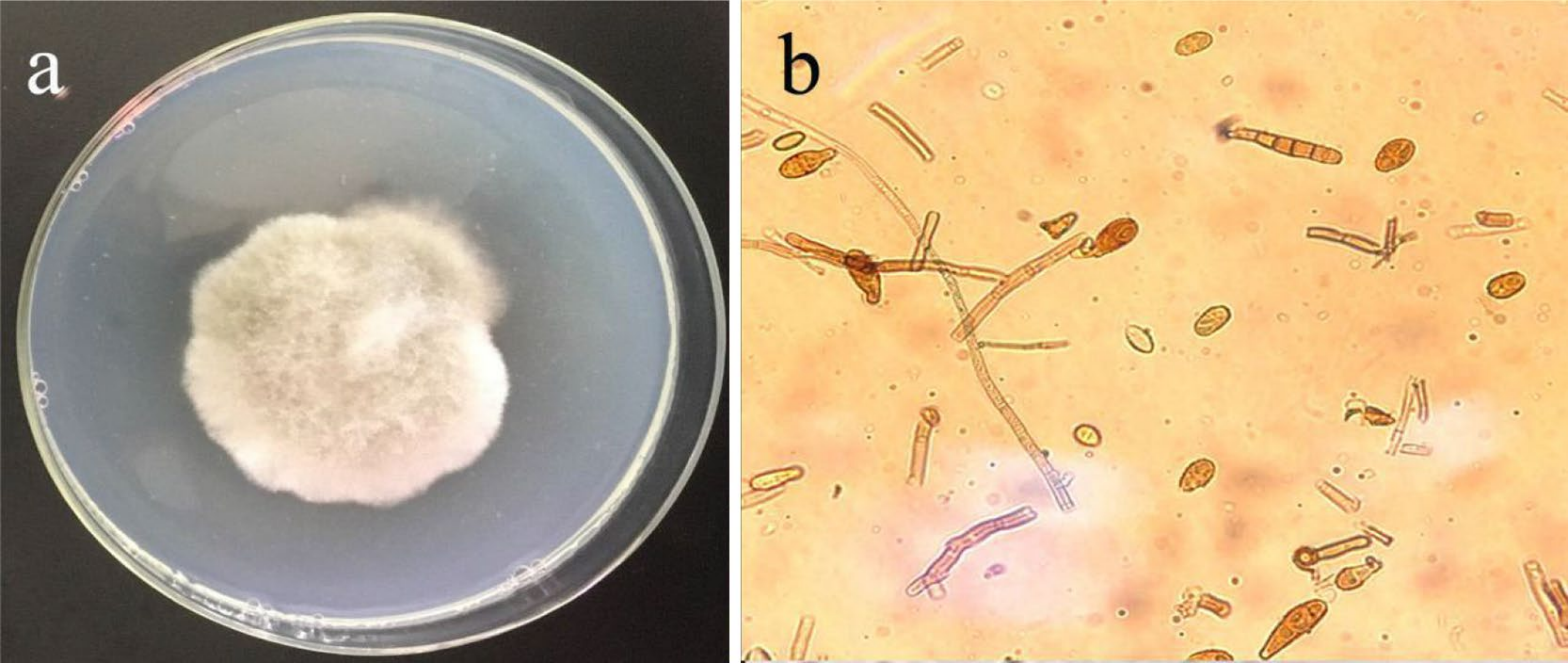
Morphological figures of *Alternaria alternata* strain Cbailin on PDA. (**a**) Colony; (**b**) Conidiophore.

Ten fungi were isolated from cultivars Kaixin666 and Berlin and then subcultured by transferring hyphal tips. Single-microconidium isolates were generated from the fungal cultures as previously described^16^ and the morphological characteristics of the isolates were analysed. Colonies on PDA medium consisted of dark grey mycelium (Fig. 1). Conidia produced after a 4-day incubation at 26 °C in darkness were pale brown to dark brown, straight or flexuous, obclavate to obpyriform or ellipsoid, and had a short conical beak at the tip or were beakless. The spores had a smooth or verrucose surface and were 3.6–11.5 μm × 6.3–35.2 μm, with 0–4 transverse septa and 0–1 longitudinal septa. Ten fungal isolates had identical morphological characteristics and were identified as *Alternaria alternata*^19^.

Genomic DNA was extracted from the single conidial cultures of two representative isolates, Cbailin and Akaixin, and the internal transcribed spacer (ITS) regions and translation elongation factor 1-alpha (*TEF-1ɑ*) genes and RNA polymerase II second largest subunit gene (*RPB2*) were amplified by PCR with primers ITS1 and ITS4^20^, EF1-728F/EF1-986R^21^ and RPB2–5F2/fRPB2–7cR^22^. A BLAST analysis revealed the amplified sequences of Cbailin and Akaixin were 100% identical to sequences from *A. alternata* isolates Alt-C81 (MN044802.1) and 13A (MK248606.1) for *ITS* and isolates Aa1 (MK733276.1) and PaF-3 (MN692926.1) for *TEF-1ɑ*, isolates 15–239 (LC132700.1) and 14–358 (LC132699.1) for *RPB2,* respectively.

**Figure 2:**
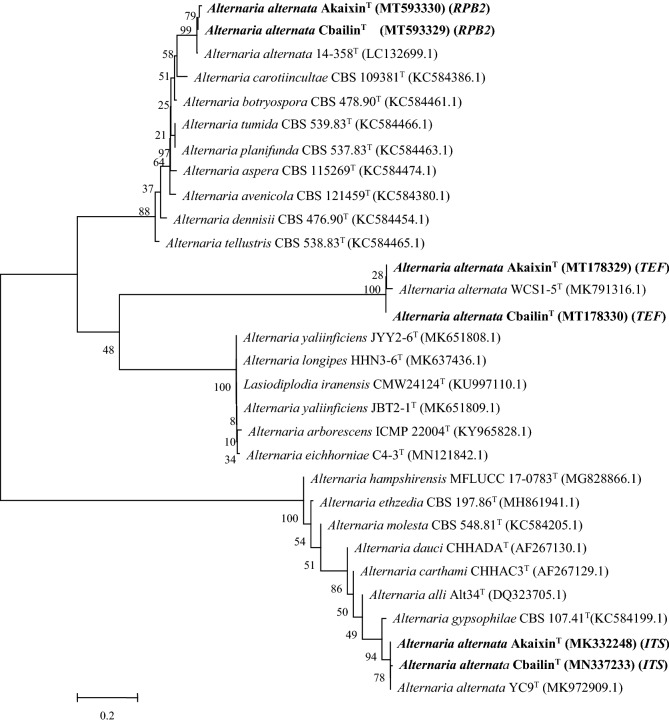
Phylogenetic tree based on the internal transcribed spacer sequence (*ITS*), translation elongation factor 1-α gene (*TEF*), and RNA polymerase II second largest subunit gene (*RPB2*) for identifying *Alternaria alternata* isolates Cbailin and Akaixin.

A combined tree based on the ITS, *TEF-1ɑ* and *RPB2* sequences indicated that Cbailin and Akaixin were *A. alternata* (Fig. 2). The sequences of the Cbailin and Akaixin amplicons were deposited in the GenBank database (accession numbers MN337233 and MK332248 for *ITS,* and MT178330 and MT178329 for *TEF-1ɑ*, and MT593329 and MT593330 for *RPB2,* respectively). To the best of our knowledge, this is the first report of the isolation of *A. alternata* from carrot seeds.

### Effects of fungal infections on carrot seed germination

The carrot seeds treated to kill all fungi germinated better than the control carrot seeds (Table 1). Specifically, the mean germination rate of seeds lacking *A. alternata* was 28.7% higher than that of control seeds infected by fungi.

**Table 1:** Effects of fungal infections on the germination of carrot seeds.

No	Treated time	Fungal inhibition rate (%)	Germination rate (%)	Reduction rate of germination (%)
1	8 h	0.0 ± 0.0a	80.0 ± 11.5b	–
	CK	47.2 ± 8.0b	56.7 ± 8.8a	29.1
2	8 h	1.3 ± 0.9a	79.8 ± 3.1b	–
	CK	50.1 ± 1.7b	59.2 ± 4.1a	25.8
3	8 h	1.0 ± 0.6a	83.1 ± 2.9b	–
	CK	50.8 ± 3.4b	57.2 ± 5.5a	31.2

Values followed by different letters were significantly different according to Duncan’s multiple range tests (*P* < 0.05), as follow tables.

^a^The treated time was 8 h, as follow tables.

^b^Values in the column indicate mean ± standard error (SE) of the carrot seed carring rate, as follow tables.

### Detection of carrot seed parts infected by fungi

**Figure 3:**
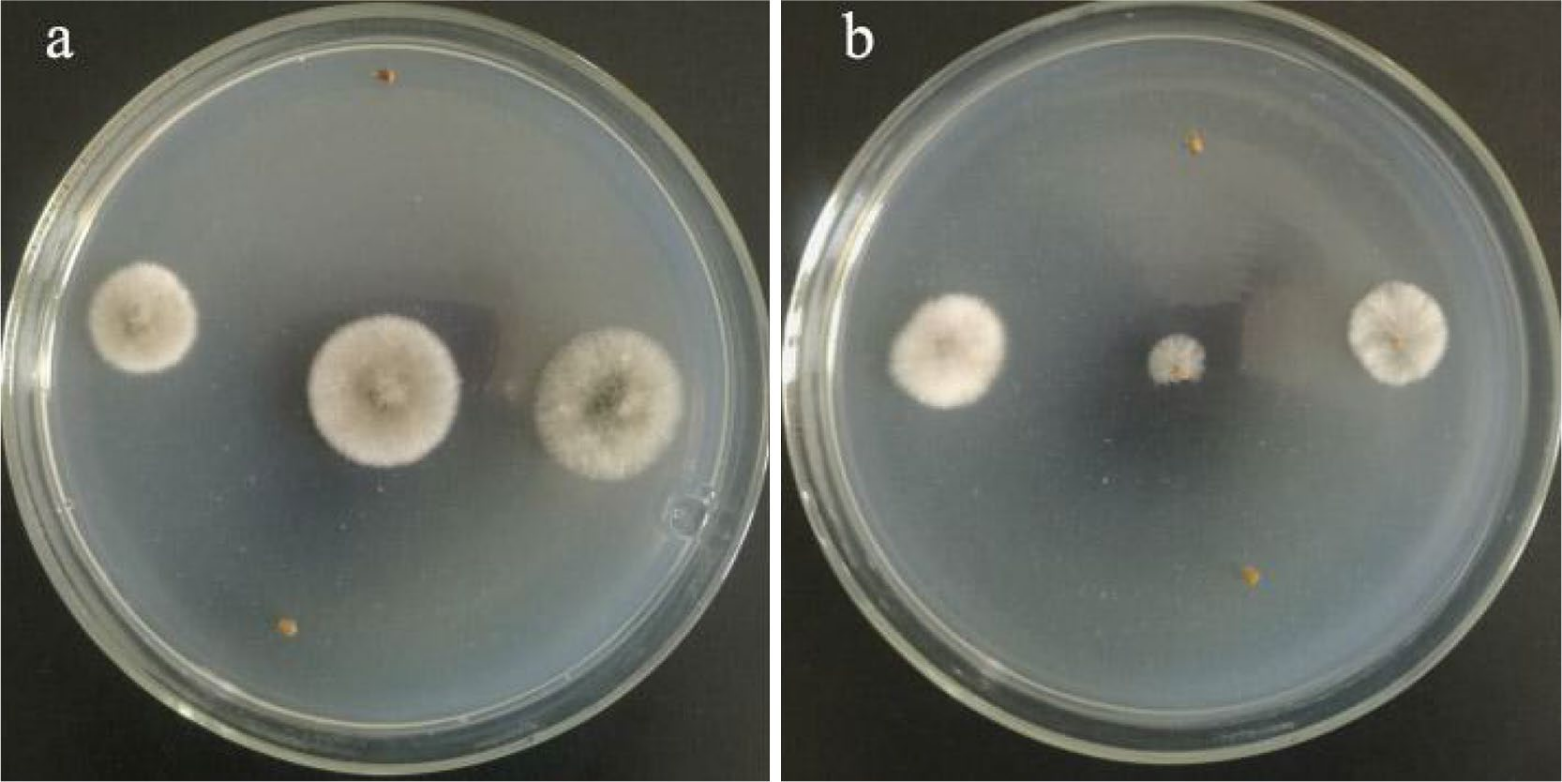
Determination of the carrot seed parts infected by fungi. (**a**) Whole seeds. (**b**) Cut seeds.

An analysis of the carrot seed parts infected by fungi revealed the seed infection rates of the whole seed and the cut seed were both 53.3% (Fig. 3). The lack of significant difference (*P* < 0.05) between the seed infection rates suggested that the fungi were just underneath the seed shell (i.e., internally seed-borne and extra-embryonic).

### Calculation of the seed infection rate of 10 carrot cultivars

Significant differences in the seed infection rates were revealed among the 10 analysed carrot cultivars (Table 2). Cultivars Hanhong and Kaixinmingtu were the most heavily infected, with seed infection rates of 60.0% and 23.3%, respectively. Second, cultivars Kaixin 1 and Bailin were very few with seed infection rates of 6.7% and 3.3%. Finally, other varieties were free of fungi.

**Table 2:** Calculation of carrot seed infection rates of various carrot cultivars.

Varieties	Carring rate	Varieties	Carring rate
Kaixinmintu	23.3 ± 6.1b	kaixin666	0.0 ± 0.0a
Zhimeihong	0.0 ± 0.0a	Hongyu6	0.0 ± 0.0a
Bailin	3.3 ± 3.3a	Kaixin838	0.0 ± 0.0a
Qiuhongyu	0.0 ± 0.0a	Kaixin668	0.0 ± 0.0a
Kaixin 1	6.7 ± 4.2a	Hanhong	60.0 ± 5.2c

### Seed treatments with various fungicides

The treatments with nine chemical fungicides resulted in considerable variability in the seed infection rate, germination, and seedling growth (Table 3). For example, amistar top SC killed all of the fungi within carrot seeds (100% control of fungal infection), whereas captan, procymidone, and azoxystrobin were less effective (40% control of fungal infection). Moreover, boscalid, difenoconazole, carbendazim, benzofuramide, and fipronil were relatively ineffective for controlling fungal infections of seeds. On the basis of the treatment effects on seed germination and seedling growth, amistar top SC was considered to be the most appropriate chemical fungicide.

**Table 3:** Effects of seed treatments with nine chemical fungicides on carrot seed infection rates, germination, and seedling growth.

Fungicides	Active ingredient	Carrying rate (%)	Control effect (%)	Plant height (cm)	Germination rate (%)	Fresh weight (g)
Azoxystrobin	250 g/L	30.0 ± 6.8b	50.0	7.1 ± 0.5ab	76.7 ± 12.0ab	0.021 ± 0.003a
Boscalid	50%	40.0 ± 7.3bc	33.3	6.1 ± 0.7a	63.3 ± 8.8ab	0.025 ± 0.003ab
Difenoconazole	10%	43.3 ± 6.1bc	27.8	6.1 ± 0.4a	60.0 ± 10.0a	0.028 ± 0.002bc
Carbendazim	80%	60.0 ± 7.3c	0.0	8.0 ± 0.2b	93.3 ± 6.7b	0.041 ± 0.003d
Procymidone	18.7%	33.3 ± 8.4b	44.4	6.8 ± 0.3a	60.0 ± 10.0a	0.029 ± 0.001bc
Sercadis plus	12%	40.0 ± 8.9bc	33.3	6.3 ± 0.3a	73.3 ± 8.8ab	0.030 ± 0.004bc
Captan	80%	33.3 ± 8.0b	44.4	7.1 ± 0.3ab	70.0 ± 10.0ab	0.023 ± 0.001ab
Fipronil	25G/L	56.7 ± 6.1c	5.6	6.8 ± 0.1a	90.0 ± 0.0ab	0.032 ± 0.001c
Amistar top SC	325 g/L	0.0 ± 0.0a	100.0	6.7 ± 0.4a	80.0 ± 5.8ab	0.030 ± 0.002bc
CK	–	60.0 ± 5.2c	–	6.4 ± 0.3a	73.3 ± 8.8ab	0.029 ± 0.002bc

### Seed treatments with varying amistar top SC concentrations

The three tested amistar top SC concentrations were inhibitory to the fungal infection of carrot seeds (Table 4). The 0.65 g/L treatment was the most effective (99.2% control of fungal infection). Additionally, seed germination and seedling growth were not inhibited.

**Table 4:** Effects of various amistar top SC concentrations on carrot seed infection rates, germination, and seedling growth.

Effective concentration (g/L)	Fungal carrying rate (%)	Control effect (%)	Germination rate (%)	Plant height (cm)	Fresh weight (g)
0.33	38.6 ± 1.9c	33.7	59.1 ± 2.6a	8.4 ± 0.1a	0.2 ± 0.0ab
0.41	11.2 ± 0.8b	81.2	72.3 ± 2.1b	8.6 ± 0.1a	0.2 ± 0.0ab
0.65	0.5 ± 0.5a	99.2	73.2 ± 2.6b	8.4 ± 0.1a	0.2 ± 0.0b
CK	59.7 ± 1.5d	–	57.9 ± 2.7a	8.2 ± 0.0a	0.1 ± 0.0a

### Effects of varying amistar top SC seed treatment times

The four tested amistar top SC treatment times inhibited the infection of seeds by fungi, with obvious differences among the analysed times (Table 5). Specifically, the 6-h and 8-h treatment times were the most effective, with no significant difference between these two time-points. However, plant height was significantly lower after the 8-h treatment. The 6-h seed treatment with amistar top SC was the most appropriate based on the overall performance.

**Table 5:** Efficacy of various amistar top SC seed treatment times.

Treated time	Inhibition rate (%)	Control effect (%)	Germination rate (%)	Plant height (cm)	Fresh weight (g)
2 h	61.1 ± 8.2ab	17.6	70.0 ± 5.8a	8.5 ± 0.1c	0.03 ± 0.00a
4 h	72.2 ± 5.5b	41.1	63.3 ± 8.8a	8.4 ± 0.1c	0.03 ± 0.00a
6 h	94.4 ± 3.5c	88.2	59.7 ± 8.8a	8.2 ± 0.0b	0.03 ± 0.01a
8 h	100.0 ± 0.0c	100.0	80.0 ± 11.5a	6.9 ± 0.1a	0.02 ± 0.00a
CK	52.8 ± 8.0a		56.7 ± 8.8a	7.9 ± 0.1b	0.03 ± 0.00a

## Discussion

Carrots are an important vegetable grown worldwide and represent a source of carotenoids in the human diet^23^. However, the emergence rate of some carrot cultivars was relatively low. The decreased germination rate was due to an infection of the seeds by *A. alternata*. The genus *Alternaria* Nees includes imperfect fungal species that are cosmopolitan and economically important. In the current study, the germination rate of seeds not infected with *A. alternata* was 28.7% higher than that of the control seeds infected with fungi. Thus, screening for suitable fungicides and optimizing their applications are important.

Seeds are critical for viable crop production. Pathogen-free seeds are essential for generating healthy plant populations and a good harvest^7^. In many crops, fungal infections are responsible for low-quality seeds^24^. Additionally, the presence of seed-borne pathogenic fungi in beans results in decreased germination, emergence, growth, and yield^25^. This is consistent with our finding that the highest carrot seed infection rate was approximately 60%. Moreover, seed infection rates differed among the tested carrot cultivars. Selecting carrot cultivars uninfected by fungi represents a good agronomic practice for minimizing the chances of fungal infections.

Microorganisms associated with seeds may be pathogens, weak parasites, or saprophytes. They may be present within or on the surface of seeds and may infect seeds via exposures to contaminated sclerotia, galls, fungal bodies, infected plant parts, and soil particles^6^. Fungal pathogens may be externally or internally seed-borne, extra- or intra-embryonic, or associated with seeds as contaminants^26^. To clarify which carrot seed parts are infected by fungi, the seed infection rates were calculated for surface-sterilized whole and cut seeds. Our results suggest the primary fungal infection site of carrot seeds is just underneath the seed shell (internally seed-borne and extra-embryonic). However, a more precise localization of the fungi is necessary.

Seeds infected by fungi influence the germination, overall health, and final crop stand under field conditions^7^. Seed-borne as well as seed-associated fungal infections can be effectively inhibited if the seeds are treated with fungicides before sowing^6^. The application of chemical fungicides can completely control fungal infections, but it can be costly and harmful to human health and the environmentyyyyy^6^. To prevent the undesirable effects of fungicides on carrots, we systematically screened for effective fungicides and examined the effects of their application. The optimal seed treatment involved a 6-h immersion in amistar top SC (effective concentration of 0.65 g/L), which killed all of the fungi infecting the seeds, with no deleterious effects on the seeds or seedlings.

The results of this study provide a theoretical basis for the development of effective methods for controlling the fungal infections of carrot seeds, thereby increasing the germination rate and vigour index.
